# Factors determining sports results in elite national volleyball teams between 2004 and 2016

**DOI:** 10.5114/biolsport.2025.139085

**Published:** 2024-07-31

**Authors:** Kazimierz Mikołajec, Barbara Nowak, Damian Banyś, Michał Krzysztofik, Dariusz Tadeusz Skalski, Alicja Markiel, Adam Maszczyk

**Affiliations:** 1 Institute of Sport Sciences, The Jerzy Kukuczka Academy of Physical Education, Katowice, Poland; 2 The Jerzy Kukuczka Academy of Physical Education in Katowice, Department of Handball and Volleyball, Mikołowska 72A, 40-065 Katowice, Poland; 3 University of Applied Sciences in Wałcz, Poland; 4 SWPS University of Social Sciences and Humanities in Katowice, Poland

**Keywords:** Volleyball, Performance variables, Regression model

## Abstract

The main objective of this study was to identify the game-related statistics determining the sports results in volleyball between 2004 and 2016. In the study, the matches of six men’s and six women’s national volleyball teams competing in the most prestigious international events were analysed. It should be emphasised that the data included in the analysis concerned the games played during the World Championship, Olympic Games, World Cup and the World Grand Champions Cup. The obtained results revealed that in the women’s group the variables number of successful attacks (p < 0.025) and number of blocking points (p < 0.044) were the fundamental factors determining success in volleyball at the elite level in the analysed period. Considering the main goal of the study, in men’s teams the number of blocking points (p < 0.007) and number of serve points (p < 0.008) were the main factors.

## INTRODUCTION

The number of volleyball players is increasing throughout the world, while the new national federations being founded provide proof of the growing popularity of this sport discipline. At present, the International Volleyball Federation (FIVB) includes 220 national sports associations, which are affiliated with five continental federations. We can expect this trend to grow even more intensively because the game continues to become more and more interesting, quicker-paced and better balanced. Both male and female players are displaying a growing level of skills. Changes in the game regulations have also affected the evolution of the players’ techniques and tactics, as well as the structure of the game. The volleyball match has been influenced the most by changes introduced by the International Volleyball Federation (FIVB) in 1998. The revised rules included changes in the scoring system, the introduction of the libero player and an extended time allowed for a serve, from five to eight seconds, with a limit of one attempt. All the changes that have been introduced into the discipline have directly affected the dynamics of the obtained results. To identify the trends in international volleyball over the past years in detail, with the possibility of simulating the results that may occur in subsequent years, it is necessary to conduct a multiple regression analysis using the function of time and significant explanatory variables. Performance factors and their relations with games’ outcomes have been a goal of scientific investigations since the 1990s. At that time Eon and Schutz [1] analysed matches from three participants of the International Volleyball Cup. Later, some studies sought to identify the relationships between selected game factors and winning matches. Some researchers have tried to predict the competition outcome using certain mathematical and statistical formulas [2, 3]. Pesaturias et al. [4] discovered that blocking was a significant factor in the game outcomes of the 2006 men’s World Championship. Silva et al. [5] found that service points, reception and blocking errors were significant factors in discriminating the possibility to predict game results and service points as variables strongly correlated with match success. However, the number of publications concerning the largest volleyball events in the world over a long period of time has remained insufficient.

## MATERIALS AND METHODS

### Research procedures

In this paper, six men’s and six women’s national volleyball teams competing in the most important international events were analysed. The study used the following variables to characterise the performance of the analysed teams:

1. attack points (successful attacks) – the AP variable;
2. attack attempts – the AA variable;
3. blocking points (successful blocks) – the BP variable;
4. blocking attempts – the BA variable;
5. serve points (successful serves) – the SP variable;
6. serve attempts– the SA variable;
7. team errors – the TE variable;
8. sports results variable – labelled SR.

The results of the following events were analysed: the World Championship games starting in 2006, the World Cups starting in 2007, the World Grand Champions Cups starting in 2005 and the Olympic Games starting in 2004 and ending with the Olympics in Rio de Janeiro in 2016.

An analysis of data obtained from the official www.fivb.org website allowed for a comparison of the best women’s and men’s volley-ball teams between 2004 and 2016.

### Statistical analysis

All variables were expressed as mean or median ± standard deviation (SD). Before using a parametric test, the assumption of normality was verified using the Kolmogorov-Smirnov test. The distributions of all variables were normal or close to normal. The numbers of quality data for analysing variables were obtained using the analysis of the contingency table.

The results and the output data were presented as mean values – entries in the table matrix. First, the values describing each variable were standardised, taking into account the number of matches played in each of the analysed years, according to the accuracy of the comparison of the variability dynamics. The totals were divided by the number of played matches, which yielded the mean values for the variables in a given year.

Because the aim during the first stage of the study was to carry out detailed and significant analyses concerning the teams’ performance and to determine the correlations between the analysed variables, a research pattern with an *RXn*_n_*Y* structure of variables was used, i.e. a single polytomous dependent variable (*Y*), as well as *n* polytomous independent variables (*Xn*_n_) in accordance with the method of non-probability sampling.

The variables describing the models of the volleyball teams that could serve as explanatory variables were established based on detailed samples and tests. The correlations between the compared test results were estimated using a univariate ridge regression analysis, for both women’s and men’s teams.

A simplified biometric model of regression was assumed with the following general form:

$$Y=\sum_{i=1}^{k}\alpha_{j}x_{j}+\xi Y$$

To determine the most important predictors of sports results, the study employed a multiple regression analysis and identified the correlations using the Pearson correlation coefficient [6, 7]. At the same time, specified variables describing the models of the volleyball teams were obtained.

## RESULTS

Results of the analysis of correlations between the variables in the groups of women’s and men’s teams and the dependent variable – the sports results – and forward ridge multiple regression analysis for the Y-SR (SR-Sport Result) dependent variable are presented below.

In Table 1, the results of the correlation analysis in the women’s group are presented. The results of the analysis of correlations between the variables and the dependent variable (sports results) in the women’s teams indicated that the AP, SP and TE variables had the strongest correlation with the SR. It can therefore be concluded that the number of attack attempts, successful serves and team errors will affect the team’s outcome.

**TABLE 1:** Pearson’s correlations between the variables describing the game structure and the sports results for the teams from Brazil, China, Japan, Russia, USA and Italy.

Brazil		China		Japan	

*Variable*	*SR*	*Variable*	*SR*	*Variable*	*SR*
AP	-0.722	AP	-0.857	AP	-0.610
AA	-0.182	AA	-0.870	AA	-0.656
BP	-0.706	BP	-0.769	BP	-0.399
BA	-0.144	BA	-0.801	BA	-0.525
SP	-0.074	SP	-0.818	SP	-0.587
SA	-0.159	SA	-0.207	SA	-0.577
TE	0.686	TE	0.222	TE	-0.568

**Russia**		**USA**		**Italy**	

*Variable*	*SR*	*Variable*	*SR*	*Variable*	*SR*

AP	-0.791	AP	-0.334	AP	-0.831
AA	-0.804	AA	-0.314	AA	-0.259
BP	-0.169	BP	-0.725	BP	-0.217
BA	-0.713	BA	-0.293	BA	-0.826
SP	-0.083	SP	-0.846	SP	-0.610
SA	-0.163	SA	-0.728	SA	-0.265
TE	0.833	TE	0.918	TE	0.202

Note: **AP** – attack points (successful attacks); **AA** – attack attempts; **BP** – blocking points (successful blocks); **BA** – blocking attempts; **SP** – serve points (successful serves); **SA** – serve attempts; **TE** – team errors.

In Table 2, the results of the correlation analysis in the group of men are presented. The correlations analysis between the variables and the dependent variable (sports results) in the men’s teams revealed that the BP, SP and AA variables had the strongest correlation with the SR. Therefore, the numbers of successful blocks, successful serves and attack attempts had the largest effect on a team’s outcome.

**TABLE 2:** Result of the analysis of correlations between the variables describing the game structure and the sports results for the teams from Brazil, Italy, USA, Argentina, Russia and Poland.

Brazil		Italy		USA	

*Variable*	*SR*	*Variable*	*SR*	*Variable*	*SR*
AP	-0.656	AP	-0.356	AP	-0.758
AA	0.727	AA	-0.713	AA	-0.324
BP	-0.632	BP	-0.618	BP	-0.673
BA	0.819	BA	-0.159	BA	-0.619
SP	-0.288	SP	-0.810	SP	-0.290
SA	-0.752	SA	0.592	SA	-0.307
TE	0.690	TE	0.580	TE	0.594

**Argentina**		**Russia**		**Poland**	

*Variable*	*SR*	*Variable*	*SR*	*Variable*	*SR*

AP	-0.208	AP	-0.286	AP	-0.596
AA	-0.297	AA	-0.622	AA	0.460
BP	-0.618	BP	-0.325	BP	-0.498
BA	-0.584	BA	0.160	BA	-0.449
SP	-0.648	SP	-0.651	SP	-0.609
SA	-0.572	SA	-0.631	SA	-0.557
TE	0.452	TE	0.278	TE	0.482

Note: **AP** – attack points (successful attacks); **AA** – attack attempts; **BP** – blocking points (successful blocks); **BA** – blocking attempts; **SP** – serve points (successful serves); **SA** – serve attempts; **TE** – team errors.

### Women

In Table 3, the results of the analysis of correlations between all the variables describing the game structure and the sports results in the women’s group are presented, while in Table 4, the results of a step-wise ridge regression analysis in the women’s group are presented.

**TABLE 3:** The results of the analysis of correlations between the variables describing game structure and sports results in the women’s group between 2004 and 2016.

All teams - women	

Variable	SR
AP	0.188
AA	-0.133
BP	-0.392
BA	-0.243
SP	0.116
SA	-0.161
TE	-0.125

Note: **AP**– attack points (successful attacks); **AA** – attack attempts; **BP** – blocking points (successful blocks); **BA** – blocking attempts; **SP** – serve points (successful serves); **SA** – serve attempts; **TE** – team errors.

**TABLE 4:** The results of a forward ridge multiple regression analysis for the SR dependent variable in the women’s group between 2004 and 2016.

Variable	Beta	B	p
Constant		4.449	0.000
AP	-2.043	-0.027	0.025
AA	1.324	0.008	0.039
BP	-0.262	-0.015	0.044

R^2 = 0.789; F = 18.190; p < .0.015

Note: **AP** – attack points (successful attacks); **AA** – attack attempts; **BP** – blocking points (successful blocks).

The forward ridge multiple regression analysis, which was conducted for the Y-SR dependent variable in the women’s group based on the determined values of the significant independent variables, led to the following form of the regression function:

$$Y-SR_{Women}=4.449-0.027\times AP+0.008\times AA-0.015\times BP$$

At the same time, the analysis revealed that the AP variable was the most important predictor of the Y-SR variable in the women’s group. The result of the regression equation means that if variable AP increases by one unit, SR will decrease by 0.027, whereas if AA increases by one unit, SR will increase by 0.008 and if SP increases, SR will decrease by another 0.015. Because no separate analysis of the attacks and counterattacks, which belonged to two different complexes (in the latest division of the technical elements), was conducted, it can be assumed that the analysed game-related statistics belonged to Complex II.

### Men

In Table 5, the results of the analysis of correlations between all the variables describing the game structure and the sports results in the men’s group are presented, while in Table 6, the results of a stepwise ridge regression analysis in the men’s group are presented.

**TABLE 5:** Results of the analysis of correlations between the variables describing the game structure and the sports results in the men’s group between 2004 and 2016.

All teams – men	

Variable	SR
AP	-0.158
AA	0.128
BP	-0.307
BA	0.098
SP	-0.349
SA	-0.012
TE	0.159

Note: **AP** – attack points (successful attacks); **AA** – attack attempts; **BP** – blocking points (successful blocks); **BA** – blocking attempts; **SP** – serve points (successful serves); **SA** – serve attempts; **TE** – team errors.

**TABLE 6:** The results of a forward ridge multiple regression analysis for the Y-SR dependent variable in the men’s group between 2004 and 2016.

Variable	Beta	B	p
Constant		3.960	0.000
AA	1.632	0.019	0.000
BP	-0.476	-0.047	0.007
SP	-1.408	-0.017	0.008

R^2 = 0.779; F = 17.190; p < .0.021

Note: **AA** – attack attempts; **BP** – blocking points (successful blocks); **SP** – serve points (successful serves).

Based on the forward ridge multiple regression analysis for the Y-SR dependent variable in the men’s group and on the determined values of the significant independent variables, the following form of the regression equation was defined:

$$Y-SR_{Men}=3.960-0.019\times AA-0.047\times BP-0.017\times SP$$

This means, for instance, that if AA increases by one unit, then the value of SR increases by 0.019. The same applies to each variable in the function, taking their signs into account.

The conducted study also showed that the AA variable was the most important predictor for the Y-SR variable in the men’s group. The result of the regression equation means that if variable AA increases by one unit, SR will increase by 0.019, whereas if BP increases by one unit, SR will decrease by 0.047, and similarly, if SP increases, SR will decrease by another 0.017. The analysis showed that, as in the women’s group, the most important technical elements affecting the sports results belonged to Complex II.

## DISCUSSION

In the first stage of the analyses conducted in this study, detailed and significant variables related to the performance and the correlations between the analysed game-related statistics were determined. The results of this research concerned the best teams in the world in the years 2004–2016 according to gender. The obtained data allowed us to identify significant determinants of sports results in elite volleyball. Among the women’s teams, the multiple regression procedure revealed a decrease in the number of attack attempts (AA), as well as an increase in the number of successful attacks (AP), and the increase in the number of successful blocks (BP) had a significant impact on the game outcome. On the other hand, correlations between the analysed variables among the men revealed that a decrease in the number of attack attempts (AA) along with an increase in the number of successful serves (SP) and blocking points (BP) showed the strongest correlation with the obtained results.

In both cases, the factors belong to Complex I and Complex II. The study did not consider a division into attacks belonging to Complex I and counterattacks belonging to Complex II. The initial analysis of variance revealed a normal distribution with slight deviations, but still within the norm [8, 9].

The results presented above do not reflect the purpose of this study in an entirely accurate manner; however, they definitely confirmed that the variables determining a victory belonged to Complex II. The top teams rely on an optimal mastery of the elements of this complex.

Similar research results were obtained by Laios and Kountouris [10] after the Olympic Games in Athens. According to their study, insufficient technical training in the elements from Complex II is one of the reasons for failure. Similar research was conducted by Castro et al. [11], who focused on the World Cup held in 2007. Their analysis encompassed game-related statistics ascribed to the appropriate complexes, while their results state explicitly that Complex II is composed of elements that, once mastered, will allow the players to carry out combination plays during a match. Castro et al. [11] also observed that in this complex, the effectiveness of an attack depends on its type, form, direction and pace, as well as on the number of blocking players. Other authors have revealed that effective defensive play allows a team to use different fast offensive options, which results in highly effective offensive play [12, 13, 29]. The results of the studies published by other authors [10, 14, 15, 16, 17, 18] indicate a successful attack as a variable that correlates with the sports results, regardless of the stage it is employed in a match. Other reports [10, 12] have confirmed the hypothesis formulated by the authors of the present study that, even if an attack does not score, it may lead to an unfavourable situation in the opponent’s defence, which can help in setting up a counterattack. Different analyses [19] have also indicated that to achieve the intended effect, a high level of effectiveness of the players in the first line of defence (block) is necessary. Others [20, 21, 29] indicate that the block is the second variable that can affect success [1, 16], which was confirmed by Afonso et al. [22] and Palao et al. [15] in their studies. The results of other research strongly indicate that offensive skills are more important in the context of the outcome of a volleyball game [12, 10, 17, 23, 24, 29, 30].

The studies by Afonso et al. [22] conducted after the Men’s World Cup in 2007 revealed that receiving a serve is a particularly important factor, as it gives the team an opportunity to use many different toss variants and a chance to score. Errors in receiving a serve will have a significant impact on the final result. However, a proper serve receive helps the team to organise an optimal attack strategy and increases the chances for a successful attack [10, 25].

The present study also involved a comparative analysis of the descriptive statistics. The results regarding the women’s teams over the period of 13 years revealed that, among all the teams, the most varied values concerned the number of attack attempts (AA), while the highest percentage variation concerned the number of serve points (SP) and the sports results (SR). Regarding the men’s teams, as with the women’s teams, the largest deviations were noted in the number of attack attempts (AA) and the serve attempts (SA), while the greatest percentage variation concerned, first and foremost, the number of successful serves (SP), the obtained sports results (SR) and the number of team errors (TE). Among both the women and the men, the distribution of the variance of the variables showed slight negative or positive skews, which nonetheless fell within the normal range [8, 9].

The obtained results suggest that when training the plans of young and senior players should predominantly emphasise the development of individual tactics in attacks. Increasing the effectiveness of this skill, particularly those from Zones Two and Four, as well as the number of attacks from the centre preceded by a good receive and an optimal, quick toss are all essential to win a volleyball game. Simultaneously decreasing the number of attack attempts and optimising the attacks will create opportunities to significantly increase the effectiveness of a team’s offensive plays. Furthermore, particular attention should be paid to the individual tactics during serves along with a number of guidelines for eliminating errors. The serve is the only element in the game of volleyball that relies on the efforts of an individual player and is crucial for obtaining a direct point. Practically every serve that scores a point requires an immediate change in the subsequent serve in order to optimise this element, thereby increasing the chance for victory.

The next stage of the study involved an analysis of the correlations between the variables and the achieved sports results. In this case, the results concerning the women’s teams showed a successful attack (AP) to be a definitive determinant of success. At the same time, the number of successful serves (SP) and avoiding team errors (TE) were both significant factors. The number of successful blocks (BP) was the least important factor. In terms of the men’s teams, the strongest correlations were observed for the serve point and the blocking point (SP and BP). The correlations identified for these variables involved a simultaneous decrease in the number of serve attempts and the attempted offensive actions (SA, AA). Conversely, the number of successful attacks (AP) showed a significant correlation with the sports results. Furthermore, the number of team errors (TE) was observed to be increasingly important. Consequently, the training practices of both men and women should include efforts to eliminate team errors. A player’s individual tactics, optimal choices and reactions, combined with perfect technical training, are highly important tools for reducing the occurrence of this variable. Executing a jump serve, particularly in the case of women, carries the risk of an error, which is why a lower number of jump serves may affect the outcome of a match. In the case of men, a successful block can also play a significant role in match outcomes. In a set, a team scores on average three blocking points; therefore, increasing this number to five is a great achievement, and while it does not guarantee success, it does increase the likelihood of winning. In order to optimise this element, it is essential to identify the individual tactics and preferences of the opposing team’s setter, as well as his or her typical behaviour towards the end of a match. All observations must be supported by working on team tactics. Similar results, which are partially confirmed by the results presented in this study, have been obtained by other authors [17, 26], who concluded that attack points and serve points are the most important determinants of sports results. Conversely, in the interpretations by Marcellino et al. [2], the attack, block and serve are considered as the elements that bring in points directly through actions, and as a result, these are what affect the final outcome. Other studies have shown that during a match between two teams playing at a similar level, the team that has committed fewer serving errors will be the winner [27], which was also confirmed by Marelica [28].

The subsequent analyses concerned the forward ridge multiple regression for the independent variables, which were conducted in order to establish the factors determining the sports results between 2004 and 2016 for each team separately while taking both the women and the men into account. Within the women’s group, the results were divided equally between successful attacks (AP) and team errors (TE), whereas in the case of the men, a successful serve (SP) was most significant. It seems, however, that such a presentation of the factors determining the sports results in volleyball is far from precise. It is definitely better to compare the determinants of the sports results for each year. Analyses of the factors involved in the women’s teams clearly indicated that the number of successful attacks (AP) and serve points (SP) were the game-related statistics with the greatest influence on the outcome of volleyball matches at the highest level. Both a successful attack and a successful serve played a decisive role in the games played during the analysed period, and the values of these variables were the highest. Therefore, it can be concluded that by increasing the number of successful attacks and serve points, the chances of achieving a victory will increase. At the same time, a successful block (BP) was found to have decreased importance.

The results of the study concerning the men’s teams, as with the women, indicated a successful serve (SP) as the most significant determinant. A successful block (BP) was found to be the second determinant, followed by a successful attack (AP). Therefore, the values of these game-related statistics (SP, BP, AP) should be supported by a lower total number of attack attempts, block attempts and serve attempts (AA, BA, SA). It was also observed that the importance of committing team errors (TE) has increased considerably in recent years, having a strong effect on the outcome of a game. It can be concluded that by increasing the effectiveness of the executed blocks and serves, with a simultaneous decrease in the number of team errors, the outcome will be affected. The publications of other authors partially confirm the above-mentioned results [5, 10, 25], indicating that a good receive of a serve allows a team to initiate a successful attack. The conducted analysis also revealed that the teams at the highest international level present a very similar level of individual and team skills, so the outcome of a match between them is typically decided by the smallest details.

## CONCLUSIONS

In conclusion, analysis of game-related statistics registered during the most prestigious international volleyball competitions during the years 2004–2016 indicates that in the case of the women’s group the numbers of successful attacks and blocking points were the fundamental factors determining success in the analysed period. Considering the men’s teams the number of blocking points and serve points had the most significant impact on the final result. Identification of these variables should contribute to appropriate planning and implementation of volleyball training.

## The approval of the Bioethics Committee for the research

The research project was approved by the Committee of Bioethics at the Academy of Physical Education in Katowice.
